# Mr. Ramsden on the Membranous Fence at the Extremity of the Urethra

**Published:** 1811-02

**Authors:** Thomas Ramsden

**Affiliations:** Coll. M. D.


*To the Editors of the Medical and Physical Journal.*

*Mr. Ramsden on the membranous Fence at the Extremity of the Urethra.*

[In answer to Mr. Foot.]

Gentlemen,

Mr. FOOT having in the Medical Journal of the last month complained of a passage in my publication on the Sclerocele, &c. as an *invasion of his claim to the discovery of a particular state of the Urethra*, I hasten through the same channel of professional information, to disavow such construction, by observing that the "*too small perforation in the glans penis*," described by Mr. Foot, and "the membranous Fence" lately adverted to by me, although somewhat alike *in principle*, are very different from each other *in appearance* and *in practical application*.

The "*too small perforation in the glans penis*,"* men-

* This state of the orifice, if I have a just conception of Mr. Foot's meaning, has no *membranous appearance*. Although it is not very frequent, yet most surgeons of experience must have met with it. Mr. Foot, tioned by Mr. Foot in his book on Vesicæ lotura, has always seemed to me to mean a small aperture, the edges of which are of equal thickness, and consequently without any *membranous* appearance. This is *treated of* by Mr. Foot *as a cause of disease to the* BLADDER *and* KIDNEYS; whereas the "*membranous fence*" or fold, described in my book on the Sclerocele, is there *treated of, as a cause of derangement to the* TESTICLE.

To shew that Mr. Foot and I mean two states of the extremity of the urethra which are different from each other, I have to remark, that in the year 1803, Mr. Foot's extensive practice, as appears from his own statement, had only supplied him with *three instances** of the "*too small perforation in the glans penis*," which could not have been the case had Mr. F. intended to speak of the same state of the extremity of the urethra as that which I have noticed under the description of a *membranous fence*—the latter being *so very common* that it will be found to exist "IN THE MAJORITY OF PERSONS *who apply for assistance under complaints of the testicle or urethra, which cannot be traced to gonorrhœal inflammation.*"

As a farther distinction I might add, that the "*too small perforation in the glans penis*," on account of the POWERFUL *resistance* it occasions to the passage of the urine or semen, becomes the cause of PAINFUL and "DANGEROUS *diseases*" of the URETHRA, the BLADDER, and the KIDNEYS, while the "*membranous fence*," on account of its occasioning *a more modified and gentle check* to the passage of those fluids, seldom induces any farther morbid affection IN THE URETHRA, than SPASMODIC STRICTURE, OR THAT PECULIAR, SUBTLE, AND SECRET DERANGEMENT of *its membrane*, which I have ascertained to be a *common cause of morbid alteration in* THE TESTICLE.

Having thus shewn that I have no wish to invade Mr. Foot's claim to the "*feather*" so fairly due to him for his practical remarks on the "*too small perforation in the glans penis*" as a cause of *dangerous diseases to the urethra, the bladder, and the kidneys*, he must pardon me if I retain

Foot, indeed, has candidly acknowledged, that in the year 1803, "*there were some among his friends who were apprised of the importance of the subject, and who treated it as he had done.*"—This fact, however, in no way takes from Mr. F.'s merit in having *so publicly demonstrated* such "too small perforation of the glans penis" to be the *cause* of *dangerous diseases of the Urethra, the Bladder, and the Kidnies.* Vide Mr. Foot on Vesicæ lotura, second part, page 84, 85, and 86, cases.

* Vide the same.

to to myself, at least until I can surrender it to a more rightful claimant, the humble credit of having directed the attention of the Profession to *another more frequent state* of the extremity of the urethra, which, although doubtless familiar to every practitioner, has to the best of my belief "HITHERTO REMAINED UNNOTICED" *as a remote cause of derangement* TO THE TESTICLE.

This, Gentlemen, is the whole and only construction I supposed could be given to the words "HITHERTO UNNOTICED", page 36 of my late *Publication* PROFESSEDLY ON THE TESTICLE, but since Mr. Foot imagines them to allow of a reading injurious to his professional fame, and it is my wish (notwithstanding Mr. F. has concluded his appeal somewhat uncourteously) to do what is proper and respectful, care shall certainly be taken when I reprint the work to free the passage from all ambiguity.

I am, Gentlemen, with respect,
Your obedient Servant,
THOMAS RAMSDEN.

*Jan.* 15, 1811. *Coll. M. D.*

